# The Warming of Hospitals

**Published:** 1901-06-29

**Authors:** Richard Greene


### EDITOR'S LETTER-BOX.

[Our Correspondents are reminded that prolixity is a great bar to publication, and that brevity of style and conciseness of statement greatly facilitate early insertion.]

### THE WARMING OF HOSPITALS.

To the Editor of *The Hospital.*

SIR,—Having just read the able review of the plans of the Victoria Hospital, Belfast, in your issue of June 22nd, and having much interest in everything connected with hospitals and asylums, I would ask your leave to supplement what your reviewer has stated as objections to the hot-air system of warming and ventilation. He truly says that equability of temperature is not, with certainty, a thing so greatly to be desired as some people imagine. It might have been added that the most delicate consumptives may inhale air of almost any degree of coldness with advantage provided the surface of the body be kept warm by sufficient clothing; indeed, this is really the guiding principle of the modern treatment of phthisis, and it is equally true of many other diseases.

Ventilation and warming by propulsion was, I believe, introduced by Desaguliers in 1734. Modifications were tried by Hales and by Arnott; but all fell into disuse, oddly enough to be revived during the last decade or so, and in some institutions the unfortunate inmates are at the present day forced to warm their bodies with the air they breathe, a system as disgusting as it is unscientific.

It is almost in vain that this system has been deprecated. Wilson says the "balance of evidence is most decidedly in favour of" extraction; and Parkes states that "for hospitals natural ventilation certainly seems the proper plan." Corfield says, "heating should be done by means of radiant heat, and not by means of air previously warmed." Leeds, of New York, writes strongly against heated air systems, and declares that "it is that dry, lifeless, withering, debilitating, poisoned stuff with which most of our houses and public buildings are filled and warmed" which is injuring the health of the American people. Stevenson says, "to make air hot enough to be the sole means of heating spoils it for breathing." More recently Dr. Jones, after much experience of the Plenum system at the large asylum at Claybury has lifted up his voice against it.

The constant breathing of this "lifeless" air is sure to cause enervation among the patients, and it is quite time that medical men protested against its introduction to hospitals as the sole means of warming. As a carefully-watched and jealously-guarded auxillary to open fireplaces, it, or some other system, is not only permissible, but may be very useful at times.

Your obedient servant,
RICHARD GREENE.

